# Gold-Coated Superparamagnetic Nanoparticles for Single Methyl Discrimination in DNA Aptamers

**DOI:** 10.3390/ijms161126046

**Published:** 2015-11-18

**Authors:** Maria Tintoré, Stefania Mazzini, Laura Polito, Marcello Marelli, Alfonso Latorre, Álvaro Somoza, Anna Aviñó, Carme Fàbrega, Ramon Eritja

**Affiliations:** 1 Department of Chemical and Biomolecular Nanotechnology, IQAC-CSIC, CIBER-BBN Networking Centre on Bioengineering, Biomaterials and Nanomedicine, C/Jordi Girona 18-26, 08034 Barcelona, Spain; maria.tintore@iqac.csic.es (M.T.); aaagma@cid.csic.es (A.A.); cgcnqb@cid.csic.es (C.F.); 2 Department of Food, Environmental and Nutritional Sciences (DEFENS), Division of Chemistry and Molecular Biology, University of Milan, Via Celoria 2, 20133 Milan, Italy; stefania.mazzini@unimi.it; 3 Department Institute of Molecular Science and Technologies, ISTM-CNR, Via G. Fantoli 16/15, 20138 Milan, Italy; laura.polito@istm.cnr.it (L.P.); marcello.marelli@istm.cnr.it (M.M.); 4 IMDEA Nanociencia & Nanobiotecnología (IMDEA-Nanociencia), Asociada al Centro Nacional de Biotecnología (CSIC), C/Faraday 9, 28049 Madrid, Spain; alfonso.latorre@imdea.org (A.L.); alvaro.somoza@imdea.org (A.S.)

**Keywords:** biosensor, gold-coated superparamagnetic nanoparticles, nanotechnology, aptamers

## Abstract

Au- and iron-based magnetic nanoparticles (NPs) are promising NPs for biomedical applications due to their unique properties. The combination of a gold coating over a magnetic core puts together the benefits from adding the magnetic properties to the robust chemistry provided by the thiol functionalization of gold. Here, the use of Au-coated magnetic NPs for molecular detection of a single methylation in DNA aptamer is described. Binding of α-thrombin to two aptamers conjugated to these NPs causes aggregation, a phenomenon that can be observed by UV, DLS and MRI. These techniques discriminate a single methylation in one of the aptamers, preventing aggregation due to the inability of α-thrombin to recognize it. A parallel study with gold and ferromagnetic NPs is detailed, concluding that the Au coating of Fe_x_O_y_ NP does not affect their performance and that they are suitable as complex biosensors. These results prove the high detection potency of Au-coated SPIONs for biomedical applications especially for DNA repair detection.

## 1. Introduction

In the last decades, a large effort has been made in the development of novel synthetic methods and functionalization of nanoparticles with biologically relevant biomolecules. This effort has led to a large number of biomedical applications such as novel diagnostic tools, delivery of drugs especially the ones that have toxic side effects, new materials for tissue engineering and new tools for molecular biology [1,2,3].

In particular, the controlled assembly of gold nanoparticles (AuNPs) has been a subject of great interest in the new field of nanobiotechnology [4,5,6,7,8,9,10,11,12]. This interest comes form the unique physical properties such as their localized surface plasmon resonance (LSPR) [13] and their efficient interaction with molecules with a free thiol group. In this way a large interest for functionalized AuNPs as potential building blocks to build nanoscale photonic and electronic devices has been developed [14,15,16,17,18,19,20,21,22]. After the description of the first DNA sensor by Mirkin *et al.* [23], the development of AuNPs-based colorimetric biosensors has been applied in the detection of most of the biomedical relevant molecular targets such as nucleic acids [17,24], proteins [15,25,26], saccharides [27], ions and small molecules [28,29] and even intact cells [30,31]. This technique takes advantage of the color changes during AuNP aggregation or redispersion events [4,5,10] and have a great potential to developed new analytical methods in the fields of analysis of environmental contaminants [28,29], clinical diagnosis [30,31], and drug discovery [32] among others.

Besides, super paramagnetic iron oxide nanoparticles (SPIONs) possess different interesting features for nanomedicine. SPIONs are well known as innovative agents in diagnostics, due to their advantages as Magnetic Resonance Imaging (MRI) contrast agents [33,34]. In comparison with the traditional contrast agents, SPIONs are less toxic, and have a strong enhancement of proton relaxation together with a low detection limit [35,36]. Furthermore, SPIONs have several other applications in biomedicine, especially for delivery purposes, due to their reduced size, the ability to be transported in biological systems [37,38,39,40,41] and the potential use for therapy by magnetic heating [42,43,44].

Gold and iron-based magnetic nanoparticles (AuSPIONs) have a prominent potential in biomedical applications due to their unique properties. The gold coating of a magnetic core combines the benefits from both nanoparticles, adding the magnetic properties to the robust chemistry provided by the thiol functionalization of the gold coating. For this reason, there is an increasing interest on the synthesis and applications of this type of gold-coated nanoparticles [39,40,45,46,47,48,49].

In this work, we describe the use of gold coated magnetic nanoparticles as molecular detection systems, through their functionalization with DNA aptamers that are recognized by the protein α-thrombin. For this purpose, we conjugated the α-thrombin binding aptamers 1 and 2 (TBA1 and TBA2), and a methylated version of TBA1 (O^6^-MedG-TBA1) (Table 1) to gold-coated iron–oxide nanoparticles, to iron-oxide nanoparticles and gold nanoparticles, in order to assess the advantages of each type of NPs. The TBA1 and TBA2 sequences bind cooperatively to specific epitopes of α-thrombin, forming a “molecular sandwich” complex [50]. TBA1 [51] is a 15 mer nucleotide and TBA2 is 29 mer [50].

**Table 1:** Oligonucleotide sequences of the three α-thrombin binding aptamers.

Name	Sequence
TBA1	HS-5’-T_15_GGTTGGTGTGGTTGG-3’
TBA2	HS-5’-T_5_AGTCCGTGGTAGGGCAGGTTGGGGTGACT-3’
O^6^-MeG-TBA1	HS-5’-T_15_GGTTG^Me^GTGTGGTTGG-3’

**Scheme 1:**
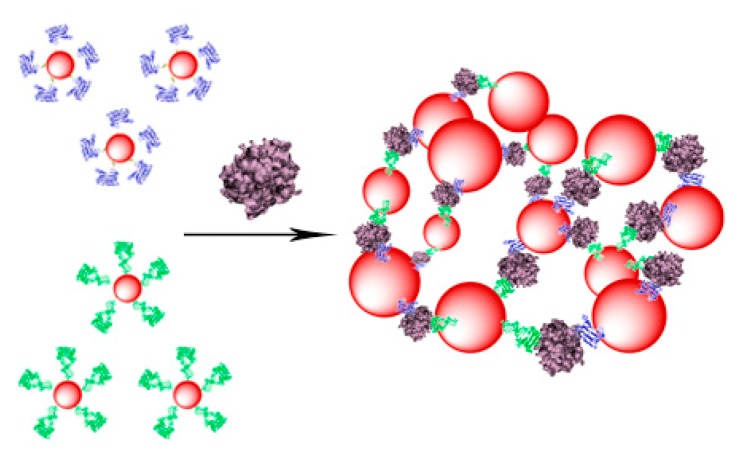
Representation of the TBA-conjugated nanoparticles and the tridimensional network formation in presence of α-thrombin for the unmodified TBAs. TBA1 and TBA2 are represented folded in their chair like structure in dark blue and in green clusters respectively α-thrombin is represented in light purple cluster.

The mixture of TBA1 and TBA2 conjugated nanoparticles should form a tridimensional network in presence of α-thrombin [41]**,** as represented in Scheme 1. This interaction can be detected in a straightforward manner using three types of techniques: Ultraviolet spectroscopy (UV), Dynamic Light Scattering (DLS) and Magnetic resonance imaging (MRI). AuSPIONs and AuNPs have a maximum of absorbance due to their surface plasmon resonance at 520 nm that shifts to higher wavelengths when aggregation occurs and this change is easily detected by UV-spectroscopy. Aggregation of the three types of nanoparticles can be detected by DLS, measuring the main hydrodynamic diameter (HD) of the nanoparticles resulting in a huge increase when α-thrombin is added to the mixture of nanoparticles carrying the TBAs. Finally, SPIONs and AuSPIONs allow the detection of the complex between α-thrombin and thenanoparticles by means of MRI, because they are contrast agents for image enhancement.

Furthermore, we also explored the discrimination capacity of the AuSPION nanoparticles to detect a single methylation in the DNA aptamer, upon destabilizing the quadruplex structure of TBA1 by the incorporation of a methyl-G in one of its tetrads. In this case, the mixture is not expected to form a tridimensional network because α-thrombin is not able to recognize this modified version of the aptamer [52,53]. Moreover, this inability to form the network allowed us to use this set of nanoparticles as a detection probe for a single methylation. This system can be further developed for the detection of the activity of DNA repair proteins, which have alkylated guanines as substrate.

## 2. Results and Discussion

### 2.1. Preparation of Gold Superparamagnetic Iron Oxide Nanoparticles (AuSPION)

**Figure 1:**
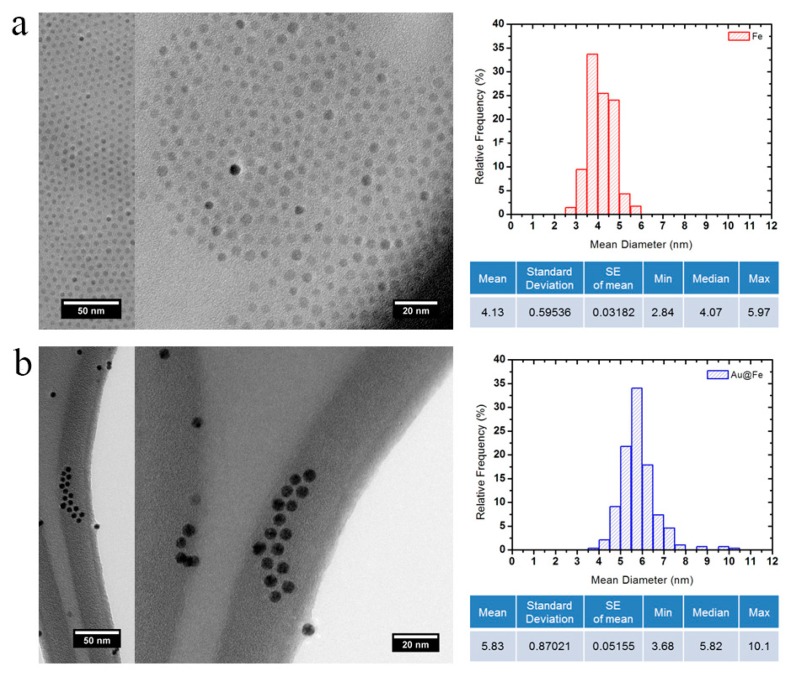
TEM images of the gold-coated magnetic nanoparticles, AuSPION. Images and particle size distribution of the ferromagnetic cores (**a**) and gold-coated SPION (**b**).

The preparation of the gold-coated nanoparticles was performed following a process consisting of two main steps, the precipitation of the ferromagnetic seeds followed by their coating with gold acetate [45,46]. This synthetic route allows the formation of homogeneous deposition of Au through a seed mediated process [45,46]. The precipitation of ferromagnetic seeds was obtained in good yield, and the resulting nanoparticle cores were characterized by TEM. TEM images (Figure 1a) show homogeneity in size and shape of the ferromagnetic core, which is an indispensable condition to achieve the final AuSPION. Then, gold acetate (source of Au^3+^) is adsorbed and reduced directly onto the NP surface to add the gold shell to the SPIONs. The purified gold-coated magnetic nanoparticles were obtained with high yield of coating and analyzed by transmission electron microscopy (TEM) (Figure 1b), showing an increase of around 1.6 nm in the core diameter, which confirms that the covering was obtained successfully. Finally, an energy dispersive X-ray analysis via scanning transmission electron microscopy (STEM–EDX) on discrete particles shows the co-presence of Au and Fe (ESI, Figure S1) along with the smooth and nearly spherical morphology [48].

### 2.2. Conjugation of the AuNPs, AuSPIONs and SPIONs with TBA1, TBA2 and O^6^-MeG-TBA1

TBA1 and TBA2, that bind to opposite sites of α-thrombin, and O^6^-MeG-TBA1 were conjugated separately and successfully to the three types of nanoparticles selected in this work.

The conjugation of TBAs to AuNPs (commercially available) and AuSPIONs was done by incubation of NPs with thiolated-oligonucleotides followed by slow salt aging as described [54].

**Figure 2:**
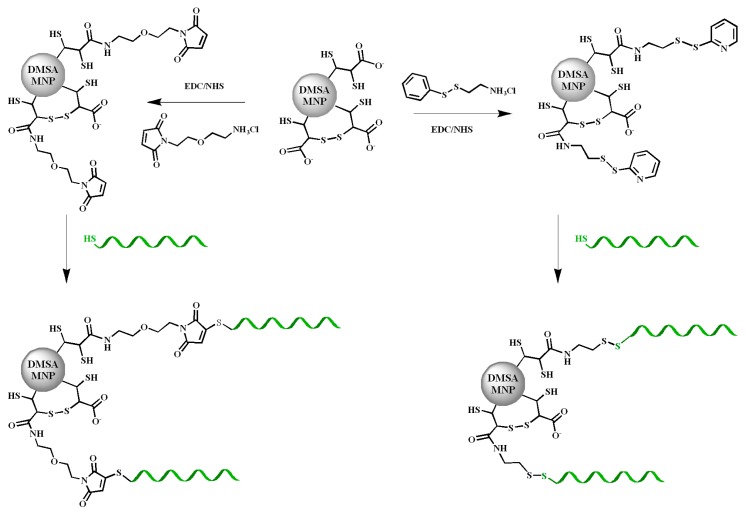
Schematic representation of the functionalization of SPIONs nanoparticles with TBAs (in green), using the two type of linkers maleimide (**left**) and disulfide (**right**).

The superparamagnetic nanoparticles were prepared as reported [55,56] and coated with dimercaptosuccinic acid (DMSA) [57]. Two type of linkers (maleimide [58] and disulfide [59], Figure 2) were used to functionalize these nanoparticles with TBAs. In order to introduce these functionalities, the carboxylate groups of DMSA were activated using EDC/NHS and then reacted with the amino groups of the corresponding linkers bearing a maleimide or disulfide moiety. The synthesis and NMR spectra of the linkers isolated as hydrochlorides are described in detail in the supplementary material section. In order to generate the free amino groups, 1 equivalent of sodium bicarbonate was used. The stability of the maleimido linker to these conditions was analyzed by ^1^H-NMR showing no significant changes in the spectrum (ESI, Figure S2). Subsequently, the resulting SPIONs were reacted with the corresponding thiolated–oligonucleotides. The degree of functionalization of SPIONs with TBAs was independent of the type of linker and they were further used indistinguishably.

The functionalization of all the nanoparticles was confirmed by the quantification of the decrease in the concentration of the oligonucleotide present in the solution, before and after conjugation (data not shown). All nanoparticles were stable after centrifugation and resuspension with aqueous buffers.

### 2.3. UV Study of the Complex Formation between α-Thrombin and AuNPs or AuSPIONs Functionalized with TBAs

The behavior of TBAs functionalized nanoparticles when incubated with α-thrombin was monitored by means of UV-spectroscopy observing the red-shift broadness of the surface Plasmon band [4,23].

**Figure 3:**
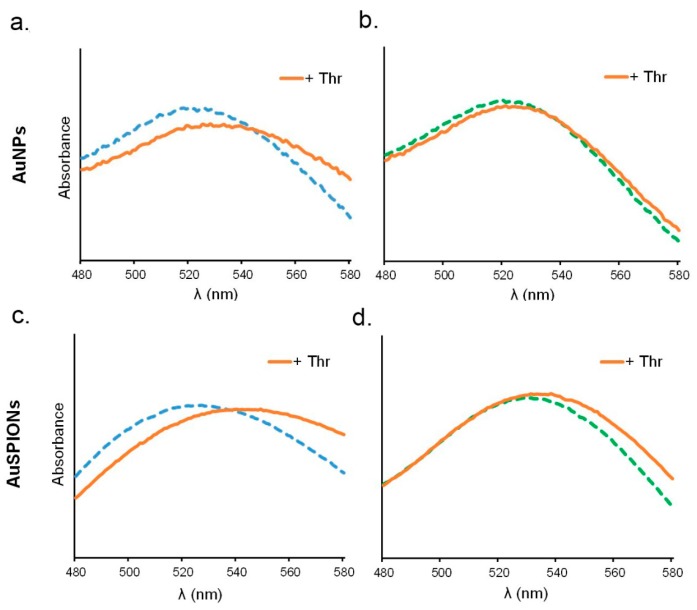
UV–Visible spectra of the complex formation between TBAs and α-thrombin at a molar ratio of (1:1). The curves represent the mixture of NPs–TBAs in the absence (dashed lines) and in the presence (orange continuous lines) of the α-thrombin. Blue represents unmodified TBAs and green, methylated TBAs: (**a**,**b**) display spectra recorded for AuNPs; and (**c**,**d**) display spectra recorded for AuSPIONs.

Binding interaction of the modified AuNPs–TBAs and AuSPIONs–TBAs with α-thrombin was carried out in phosphate buffer with additional K^+^ at 25 °C. As expected, the UV spectrum for TBA1 and TBA2 anchored to AuNPs or AuSPIONs showed a maximum of absorption at 520 nm before incubation with α-thrombin (Figure 3). This maximum was displaced to higher wavelengths in a continuous way when α-thrombin was added, until reaching stabilization (30 nm). The UV spectra of these titrations are shown in Figure S3 (ESI).

These results confirmed that α-thrombin was interacting with both TBA1 and TBA2, creating a NP network formed by the binding of thrombin to both TBA1 and TBA2 sequences. In addition, the formation of this network of interactions between α-thrombin and TBAs is not affected by the nature of the nanoparticles, as both AuNPs and AuSPIONs showed the same response with a detection limit in the 125 to 250 picomolar range.

In contrast, the interaction between α-thrombin and the mixture of O^6^-MeG-TBA1 and TBA2 anchored to the two types of nanoparticles (AuNPs or AuSPIONs) was evidently smaller. As it can be seen in Figure 3b,d, at equal concentrations of α-thrombin the maximum of absorption was not changed in AuNPs (Figure 3b) or slightly displaced in Au SPIONs (Figure 3d). This small difference in the displacement rate observed in Au SPIONs (less than 10 nm) can be explained by the only interaction of AuNPs–TBA2 or AuSPIONs–TBA2 with α-thrombin. These observations proved that α-thrombin was not able to form the network with the same performance as in the case of the unmodified TBAs, and that its *K*_D_ is much higher when one guanine in the central tetrad of the TBA sequence is modified with a methyl group in the O^6^ position. This simple modification prevents the formation of its quadruplex structure, and for this reason, it cannot be recognized by α-thrombin. Moreover, when we forced the concentration of α-thrombin *versus* TBAs (molar ratio 4:1), we could observe a small displacement of the maximum in the UV spectrum of the mixture containing the methylG-TBA1 conjugated with both types of NPs. This effect could be due to the formation of small clusters, even if in a reduced way if compared to the non-methylated mixture of TBAs (10 nm for AuSPIONs and 15 nm for AuNPs). As the protein itself is able to recognize the sequence of methylated TBA and force it to fold in its quadruplex structure suitable for the binding [52], we presume that the methylated sequence is also recognized as a result of the excess of protein in the mixture. It is interesting to note that α-thrombin attempts to fold it and this may result in some degree of binding.

UV seems a proper and simple way to measure the interaction between α-thrombin and the TBAs nanoparticles, as the change can be visualized within a direct step and it is not cost nor time consuming. This result confirmed that macromolecular aggregation processes can be studied indistinguishably by these two types of nanoparticles, demonstrating that the gold coating maintains the chemical and optical properties of gold itself.

### 2.4. DLS Study of the Complex Formation between α-Thrombin and NPs-TBAs

**Figure 4:**
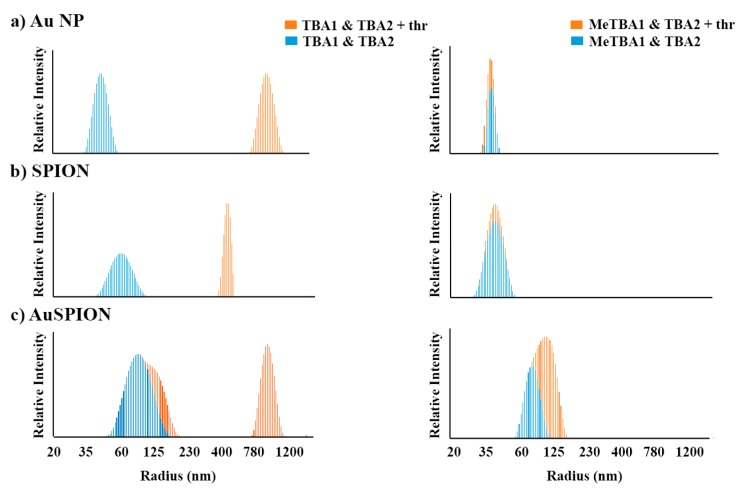
Particle size distribution of NPs-TBAs at 25 °C recorded by DLS. The mixtures of TBA1 and TBA2 NPs (**left**) and O^6^−MeG-TBA1 and TBA2 NPs (**right**) are displayed in dashed blue, while the same NP mixtures in the presence of α-thrombin are represented in orange. Each image is the result of the combination of two DLS experiments with and without α-thrombin. The individual DLS measurements are shown in Figure S4 (ESI). In all cases, the molar ratio of α-thrombin: TBAs was 0.5:1. From top to bottom: (**a**) AuNP, (**b**) SPIONs and (**c**) AuSPION.

The study of the complex formation between the three types of NPs–TBAs and α-thrombin by dynamic light scattering was carried out in phosphate buffer with additional K^+^ at 25 °C. Figure 4 and Table 2 show the particle size distribution for the mixture of the different types of TBAs nanoparticles with and without α-thrombin. Particle size analysis was performed after the addition of α-thrombin into a mixture of TBA1 and TBA2 anchored to the three different types of nanoparticles (AuNPs, SPIONs and AuSPIONs). In all three cases the average diameter of the nanoparticles immediately increased by 25-, 21.5- and 7-fold (see Table 2). Even if a small polydispersity is observed in the initial state of the mixture with AuSPION, we can clearly detect the formation of the α-thrombin-TBAs network with a detection limit of 50 pmols of DNA No precipitation was observed at these complexes concentration during the measurements. Similar results were observed for the SPIONs independently of the linker used for the functionalization.

**Table 2:** Size distribution (HD, nm) by DLS of TBAs nanoparticles in the absence and in the presence of α-thrombin.

Nanoparticles Type		Without α-Thrombin HD (nm)	With α-Thrombin HD (nm)
AuNPs	TBA1 & TBA2	38 ± 5	947 ± 283
	O^6^-MeG-TBA1 & TBA2	38 ± 5	56 ± 7
SPIONs	TBA1 & TBA2	36 ± 5	781 ± 185
	O^6^-MeG-TBA1 & TBA2	39.5 ± 11	39.5 ± 11
AuSPIONs	TBA1 & TBA2	91 ± 25	633 ± 225
	O^6^-MeG-TBA1 & TBA2	91 ± 25	109 ± 34

In contrast, the interaction between equal concentrations of α-thrombin with the mixture of each one of the three types of O^6^-MeG-TBA1 and TBA2 nanoparticles was inexistent or clearly smaller (see Table 2). These observations indicate that α-thrombin was not able to form the network with the same efficiency when the TBA1 was substituted by methylated TBA1, and thus the particle size average remained similar. In these cases, the interaction between NPs–TBA2 and α-thrombin was not identified. We presume that this may be due to the small variation in the size of the nanoparticles, which is too low to be observed by DLS.

### 2.5. Magnetic Resonance Imaging

The study of the complex formation between α-thrombin and the TBA aptamers by magnetic resonance imaging was already been described by Yigit *et al.* [41]. In our work we went a step forward in the application of the MRI technique using gold-coated magnetic nanoparticles as contrast agents, studying also the effect produced in the MRI contrast when TBA1 is substituted by a methylated TBA1.

A 1:1 mixture of TBA1 or O^6^-MeG-TBA1 and TBA2 anchored to SPIONs or AuSPIONs was prepared in phosphate-K^+^ buffer at 25 °C. Table 3 summarizes the formation of the molecular network due to α-thrombin binding to the nanoparticles, monitored by the changes in brightness of the T2-weighted MR images. A 2.4 µg Fe/mL concentration of the SPIONs resulted in a T2 of 50 ± 3 ms with a detection limit in the low picomolar range. After aggregation of the mixture by adding α-thrombin, the magnetic relaxation properties changed, reducing the T2 relaxation time to 40 ± 3 ms. Even if the concentration of the AuSPIONs nanoparticles was required to be 1000 times higher, due to the fact that the gold coating decreases the magnetic response, a similar reduction in the T2 was observed. Again, the T2 values for the mixture containing methylG-TBA1 did not decrease when α-thrombin was added, indicating that the network could not be formed. These observations are in agreement with our previous results obtained by UV and DLS studies, and prove that gold-coated magnetic NPs can be used for MRI detection of biomolecules.

**Table 3:** T2 relaxation times in the absence or presence of α-thrombin. SPIONs were measured at 1/1000 (2.4 µg Fe/mL) and the AuSPIONs 1/1 (2.4 mg Fe/mL). These results represent the average of at least three independent measurements.

Nanoparticles Type		Concentration of α-Thrombin	
		0	5 nM
SPIONs	TBA1 & TBA2	50 ± 3 ms	40 ± 3 ms
	O^6^-MeG-TBA1 & TBA2	51 ± 4 ms	51 ± 5 ms
AuSPIONs.	TBA1 & TBA2	70 ± 1 ms	62 ± 1 ms
	O^6^-MeG-TBA1 & TBA2	63 ± 2 ms	62 ± 1 ms

## 3. Experimental Section

### 3.1. Chemicals

Human α-thrombin was purchased from Heamatologic Technologies Inc (Cambridge, UK). All reagents and dry solvents were from Sigma-Aldrich (Tres Cantos, Spain) or Fluka (Sigma-Aldrich Quimica S.A, Alcobendas, Spain). Other chemicals from specific commercial source will be specified as they are named. Standard phosphoramidites were purchased from commercial sources (Applied Biosystems, Foster City, CA, USA). Oligonucleotide synthesis reagents were from ABI (Foster City, CA, USA). Gold nanoparticles (10 nm) stabilized in aqueous solution of citrate were purchased from Sigma-Aldrich. Low binding eppendorf tubes were used in the preparation of the nanoparticles in order to avoid adsorption to the tube. The matrix for MALDI-TOF measurements had 2’,4’,6’-Trihydroxyacetophenone (THAP, Sigma-Aldrich Tres Cantos, Spain) and ammonium citrate (Fluka).

### 3.2. Instrumentation

RP-HPLC was performed as described [52]. Oligonucleotide concentration and UV spectra were measured on a JASCO V-650 instrument. Mass spectra were recorded on a MALDI Voyager DE™ RP time of-flight (TOF) spectrometer (ABI, Foster City, CA, USA). Dynamic light scattering (DLS) studies were performed on a DLS spectrometer (LS Instruments, Fribourg, Switzerland) equipped with a He-Ne laser (Brookhaven Instruments Corporation, Holtsville, NY USA, 632.8 nm). MRI measurements were performed on a Bruker Avance AV600 spectrometer (Bruker Biospin, Rheinstetten, Germany) equipped with a 10 mm 1H micro-imaging probe and a variable-temperature control unit. Acquisition and data processing were done using ParaVision v. 4.0 (Bruker BioSpin MRI, Rheinstetten, Germany). TEM analysis was performed on a Transmission Electron Microscope (TEM) LIBRA^®^ 200FE (Carl Zeiss AG, Jena, Germany) with an electron beam source of 200 keV, a resolution power of 0.24 nm and a magnification of 8×–1,000,000×.

### 3.3. Oligonucleotides Synthesis

The three oligonucleotide sequences (Table 1) were prepared on 200-nmol scale synthesis using an ABI 3400 DNA Synthesizer (ABI, Foster City, CA, USA). The preparation of thiolated sequences at the 5’-end implied the use of 5’-thiol modifier C6 S–S phosphoramidite (Link Technologies, Glasgow, Scotland, UK). For strands containing O^6^-MeG, we used G^DMF^ phosphoramidite. O^6^-MeG-TBA1 was deprotected in ammonia solution, overnight at room temperature. The resulting products were desalted on NAP-10 columns (Amersham Biosciences, Barcelona, Spain) and analyzed by reversed-phase HPLC. All the products had the expected mass by MALDI-TOF. HO(CH_2_)_6_–S–S–(CH_2_)_6_–T_15_TBA1 [*M*] = 9617.1 (expected 9612.6), HO(CH_2_)_6_–S–S–(CH_2_)_6_–T_15_–O^6^–MeG–TBA1 [*M*] = 9621.4 (expected 9626.6), HO(CH_2_)_6_–S–S–(CH_2_)_6_–T_5_–TBA2 [*M*] = 10795.5 (expected 10797.9).

### 3.4. Synthesis of SPIONs

DMSA coated Fe_x_O_y_ nanoparticles were obtained in good yield through thermal decomposition, following the protocol reported by Salas *et al*. [55]. Resulting NPs show superparamagnetic properties and are active in MR imaging. According with the synthetic methodology, the material is composed by a mix of magnetite (Fe_3_O_4_) and maghemite (γ-Fe_2_O_3_) [60]. Due to that, the SPIONs are labelled as Fe_x_O_y_.

### 3.5. Synthesis of Maleimide Linker

2-(2-Aminoethoxy)ethanol (2 mL, 18 mmol) was protected with (BOC)_2_O in THF, followed by a standard workup. The resulting protected compound was left to react with maleimide (2.1 g, 22.5 mmol) in THF, and a freshly solution of PPh_3_ (3.6 g, 13.5 mmol) followed by DIAD (3.6 mL, 18 mmol) as described [58]. The product was precipitated in Et_2_O, filtered and purified by flash chromatography (Hexane/AcOEt 5:2 to 1:1). Then, it was deprotected with trifluoroacetic acid and purified to obtain a final yield of 89%. The resulting product had the same physical and spectroscopical properties as the one described by Weber *et al*. [61]. See Supplementary Materials for more detailed description of the synthesis and characterization of these products (see Figure S5).

### 3.6. Synthesis of Thiopyridinyl Linker (PDA*HCl)

The thiopyridinyl linker was obtained following reported protocols [59]. Briefly, to a solution of aldrithiol (213 mg, 0.96 mmol) in MeOH, 2-mercaptoethylamine hydrochloride (109 mg, 0.96 mmol) was added. After 1hr of stirring, the reaction was stopped by evaporation of the solvent and the residue was washed with cold AcOEt three times. PDA*HCl was obtained in 51% yield (white solid). ^1^H-NMR (300 MHz, CD_3_OD) δ 8.57 (d, 1H, *J* = 5.0 Hz), 7.83 (t, 1H, *J* = 7.7 Hz), 7.69 (d, 1H, *J* = 8.0 Hz), 7.35 (dd, 1H, *J* = 7.5, 5.0 Hz), 3.18 (t, 2H, *J* = 6.1 Hz), 3.07 (t, 2H, *J* = 6.8 Hz).

### 3.7. Functionalization of DMSA Coated Fe_x_O_y_ Nanoparticles with Maleimide or PDA*HCl Linker

To 1 mL of SPIONs at 2.4 mg Fe/mL, the maleimide or PDA*HCl linker (50 µmol/g Fe), 1 equivalent of NaHCO_3_, 150 µmol of WSC(EDC)/g Fe and 75 µmol of NHS/g Fe were added. The suspension was stirred at R.T. overnight. The resulting NPs were washed by iterative centrifugation and redispersion in milliQ water for at least 3 times.

### 3.8. Synthesis of AuSPIONs

#### 3.8.1. Synthesis of Fe_x_O_y_ MNP as Seeds

First, 0.355 g of iron (III) acetylacetonate (1 mmol) were dissolved in phenyl ether (10 mL) with oleylamine (1 mL, 2 mmol) and oleic acid (1 mL, 3 mmol) in inert (Ar) atmosphere and vigorous stirring. Then, 1.29 g of 1,2-hexadecanediol (5 mmol) were added and the resulting mixture was heated under reflux for 2 h at 210 °C. Finally, the resulting suspension was left to cool down to R.T. [45]. The final solutions were used in the following steps without further purification.

#### 3.8.2. Reduction of Au–Acetate (Coating) 

First, 2.5 mL of the Fe_x_O_y_ nanoparticles solution previously prepared (approximately 0.166 mmol Fe_x_O_y_) were diluted to a final volume of 7.5 mL in phenyl ether and mixed up with Au(OOCCH_3_)_3_ (0.55 mmol, 0.208 g), oleic acid (0.375 mmol, 0.125 mL), oleylamine (1.5 mmol, 0.75 mL) and 1,2-hexadecanediol (3 mmol, 0.775 g), under Ar atmosphere and strong stirring. Then, the temperature of the solution was increased following a ramp of 10 °C/min until reaching 190 °C, and the suspension was refluxed for an extra 1.5 h. Then, AuSPIONs were precipitated with ethanol and purified by centrifugation. The precipitate was washed twice and redispersed in a 1 M TMAOH solution. Then, 0.04 g of trisodium citrate were carefully added adjusting the pH of the solution to 6.5. Finally, the solution was sonicated for 30 min, and the nanoparticles were collected using a magnet and redispersed in pure water [46,47].

### 3.9. Functionalization of the Different Type of Nanoparticles

#### 3.9.1. Gold Nanoparticles (AuNPs)

First, 2–3 ODs of TBA1, -TBA2 and O^6^-MeG-TBA1 were reduced with 300 µL TEAAc 0.1 M and TCEP·HCl 34 μL (100 mM) at 55 °C overnight, to prevent the formation of disulfide bridges and to break the already existing ones. Next, 1 OD of the deprotected oligonucleotides were diluted in 0.5 mL of MilliQ water and desalted (NAP-5 column). The resulting solution (1 mL) was mixed with the AuNPs solution (1 mL) and left to conjugate overnight under agitation. Then, the solutions were adjusted to 10 mM Na_x_PO_4_H_y_ (pH = 7.0). The resulting mixtures were allowed to equilibrate and then they were brought to a concentration of 0.15 M sodium chloride stepwise and over a period of 9 h. The solutions were treated in a sonicator for 10 s before every addition in order to keep nanoparticles dispersed. Then, the suspensions were incubated overnight at room temperature. At the end, the nanoparticles were purified by centrifugation at 13,200 rpm (16,100× *g*) using a buffer containing 0.15 M sodium chloride, 10 mM Na_x_PO_4_H_y_ (pH = 7) and NaN_3_ 0.01%. The centrifugation process was performed 4 times for 45 min at 15–20 °C. The TBAs-conjugated gold nanoparticles were analyzed by UV–visible absorption. TBAs conjugated nanoparticles were stored at 4 °C and sonicated during 5 min before use.

#### 3.9.2. Superparamagnetic Iron Oxide Nanoparticles (SPION)

Aliquots of thiolated-aptamers (TBA1, TBA2 and O^6^-Me-TBA1) for a final concentration of 245 µM were mixed with 200 µL of 2.4 mg Fe/mL Fe_x_O_y_ magnetic nanoparticles functionalized with maleimido and 2-thiopyridinyl groups [57] and left overnight at R.T. The TBA-conjugated nanoparticles were purified by iterative centrifugation-resuspension process. The centrifugation was repeated 3 times for 30 min at maximum speed at room temperature and with the addition of small amounts of NaCl to enhance precipitation if required. The covalently immobilized TBA was determined by quantification of the unbound TBA in the collected supernatants. The TBAs conjugated nanoparticles were analyzed by UV–visible absorption and stored at 4 °C until further use.

#### 3.9.3. Gold Superparamagnetic Iron Oxide Nanoparticles (AuSPION)

Equal volume of gold coated nanoparticles and thiolated DNA solution (1 OD) were mixed and left to conjugate under agitation and at R.T. for 48 h. The resulting mixtures followed the same salt aging process than AuNPs and the resulting suspension was centrifuged under the same conditions used for the AuNPs, resuspended in MilliQ water to remove non-conjugated oligonucleotides, and observed for any indication of aggregation. Finally, the conjugated oligonucleotides gold coated superparamagnetic nanoparticles were analyzed by UV–visible absorption. The conjugated nanoparticles were kept at 4 °C in MilliQ water until further use, due to precipitation when adding salts to the solution.

### 3.10. Studies of α-Thrombin Interactions with TBA-Functionalized AuNPs and AuSPIONs by UV

The interactions between *α*-thrombin and TBA1, TBA2 and O^6^-MeG-TBA1 nanoparticles were monitored by measuring changes in the UV spectrum (recorded from 650 to 400 nm) upon increasing concentrations of the protein (125 nM up to 1 µM). A mixture of NP–TBA1 and NP–TBA2 were diluted in buffer containing 10 mM phosphate pH 7 and 5 mM KCl to reach a concentration of 5 nM of nanoparticles in a volume of 500 µL. As conjugation was approximated to represent a coating of 100 oligonucleotides per nanoparticle, the concentration of DNA in the sample was considered to be around 500 nM. Each time, enough quantity (125 nM) of human α-thrombin was added. After a quick manual mix, the UV spectra were recorded. The UV spectra of the mixture and increasing concentrations of α-thrombin was recorded until reaching a final concentration of 1 µM, which represents a 2:1 molar ratio between α-thrombin and DNA. This same experiment was repeated using the modified NP–methyl TBA1 and NP–TBA2. All spectra were overlaid together to study the displacement of the maximum peak from 520 nm in case of AuNPs and 545 nm for AuSPIONs to higher wavelengths, due to the formation of a network of TBAs-α-thrombin complexes. Negative controls have been carried on by using TBA1, TBA2 and O^6^-MeG-TBA1 along with increasing concentrations of hAGT and scrambled DNA along with α-thrombin. The protocol used for these studies was the same for AuNPs and for AuSPIONs.

### 3.11. Studies of α-Thrombin Interaction with TBAs Nanoparticles (AuNPS, SPIONs and AuSPIONs) by DLS

Binding between α-thrombin and TBA1, TBA2 and O^6^-MeG-TBA1 nanoparticles was studied by DLS measuring the average HD of the TBA-functionalized particles at R.T. The molecular network originated from this binding was monitored by measuring the particles’ radius enlargement upon increasing concentrations of the protein. One hundred microliters of a 5 nM mixture of NP–TBA1 and NP–TBA2 in buffer containing 10 mM phosphate (pH = 7) and 5 mM KCl were measured during 100 s using a 3D cross method with a scattering angle of 90° at 25 °C. The final concentration of the TBAs in the samples was 500 nM. Enough quantity of human α-thrombin was added to reach a 0.5:1 molar ratio between α-thrombin and DNA. After a quick manual mix, the radius length was recorded. The same experiment was repeated using the modified NP–O^6^-MeG-TBA1 and NP–TBA2. The particle radius was measured by adjusting the first cumulant parameter. The relative intensity of the different particle complexes was measured by computer fitting the contin parameter. All the experiments were performed in triplicates. The procedure of the DLS measurements was the same for AuNPs, SPIONs and AuSPIONs.

### 3.12. Studies of α-Thrombin Interaction with TBAs Nanoparticles (SPIONs and AuSPIONs) by MRI

NPs–TBA1 (or NPs–O^6^-MeG-TBA1) and NPs-TBA2 were mixed in 1:1 ratio. Then, the mixture was diluted in 100 mM sodium chloride, 25 mM potassium chloride and 25 mM tris-HCl at pH = 7.4. The final concentration of nanoparticles was 2.4 µg Fe/mL in the case of the SPIONs and 2.4 mg/mL for the AuSPIONs. Then, the samples were incubated with different concentrations (0, 5, 10 and 20 nM) of α-thrombin. One hundred microliters of the mixed sample were loaded into a microcapillar and T2 experiments were recorded. The magnetic field strength was 14 T. This corresponds to a 1 H resonance frequency of 600.1 MHz. The images of the solutions were acquired at R.T. We used the following parameters for the acquisition of data: number of slices: 1; MSME (Multi Slice Multi Echo) acquisition; thickness: 1.50 mm; echo time: 4.5 ms; repetition time: 1500 ms; number of echoes: 10; FOV: 0.8 mm. We performed two scans. This corresponds to a total acquisition time of 12 min. T2 values were extracted using a non-linear multi-parametric fitting *y* = A + B_e_ − *t*/*T*_2_ of the intensity decays. All measurements were performed by triplicate of four independent experiments. The MRI measurements of the AuSPION were performed following the same protocol as the one used for the SPION. Acquisition and data processing were performed with ParaVision v. 4.0 (Bruker BioSpin MRI, Rheinstetten, Germany).

## 4. Conclusions

We have obtained in high yield and purity gold-coated magnetic nanoparticles. Their functionalization with oligonucleotides is comparable with the one obtained for the gold nanoparticles. Our results prove the capacity of AuSPIONs to detect a protein by three diverse techniques. AuSPIONs were suitable for UV experiments with the same performance as AuNP, without major changes in the execution nor in the detection limit. AuSPIONs showed similar behavior in DLS measurements to AuNPs and SPIONs, resulting in very similar plots of size distribution. Finally, AuSPION were useful for MRI experiments, even if they showed a shielding of the magnetic resonance due to the gold coating and required higher concentrations to reach the same signal as SPIONs.

Furthermore, this work proves that AuSPION, as well as AuNPs and SPIONs, can discriminate a native TBA from a methylated one in the analysis of the complex formation between TBAs and α-thrombin by biophysical methods. As O^6^-methylguanine is the substrate for DNA repair enzymes, critical for chemotherapy resistance, this method can also be used to monitor the activity of these DNA repair proteins. Different detection methods for the evaluation of the repair activity of one of them, alkylguanine–DNA-transferase (hAGT) [62] have been previously described [52,53]. These methods are also based on the conformational change of TBA upon methylation of a single guanine. Its repair restores the G-quadruplex and its recognition by α-thrombin. We envisage that AuSPIONs are useful for the development of a new methodology to detect hAGT activity in a straightforward manner. Work in this direction is currently ongoing in our laboratory. We believe that the multidisciplinary detection capacity of AuSPIONs can be evolved to design more complex biosensors for biomedical applications as well as for drug delivery and *in vivo* imaging.

## Supplementary Materials

Supplementary Materials are available online at www.mdpi.com/1422-0067/16/11/26046/s1.

## Abbreviations

AuNPs: gold nanoparticles, AuSPIONs: gold-coated superparamagnetic iron oxide nanoparticles, DLS: dynamic light scattering, DMF: dimethylformamidino group, DMSA: dimercaptosuccinic acid, DTT: dithiothreitol, Fe_x_O_y_: iron oxide, hAGT: human O*^6^*-alkylguanine–DNA alkyltransferase, HD: Hydrodynamic diameter, HPLC: high performance liquid chromatography, LSPR: localized surface plasmon resonance, MRI: magnetic resonance imaging, NHS: *N*-hydroxysuccinimide, NPs: nanoparticles, O^6^-MeG: O^6^-methylguanine, O^6^-MeG-TBA1: O^6^-methylguanine α-thrombin-binding aptamer, R.T. room temperature*,* SPIONs: superparamagnetic iron oxide nanoparticles, T2: spin-spin relaxation time, TBA: α-thrombin-binding aptamer, TCEP·HCl: tris(2-carboxyethyl)phosphine hydrochloride, TEAAc: triethylammonium acetate, TEM: transmission electron microscopy, TMAOH: tetramethylammonium hydroxide, Tris: Tris(hydroxymethyl) aminomethane, WSC water soluble carbodiimide (also abbreviated as EDC: 1-ethyl-3-(3-dimethylaminopropyl)carbodiimide.
