# Supplementary material for: Gold-Coated Superparamagnetic Nanoparticles for Single Methyl Discrimination in DNA Aptamers
Source: Int J Mol Sci. 2015 Nov 18;16(11):27625–39. doi: 10.3390/ijms161126046 (PMC4661904; doi:10.3390/ijms161126046)
Supplement: Supplementary file 1 [file ijms-16-26046-s001.pdf]

Supplementary Materials: Gold-Coated Superparamagnetic Nanoparticles for Single Methyl Discrimination in DNA Aptamers

Maria Tintoré, Stefania Mazzini, Laura Polito, Marcello Marelli, Alfonso Latorre, Álvaro Somoza, Anna Aviñó, Carme Fàbrega and Ramon Eritja

1. Synthesis of maleimide linker

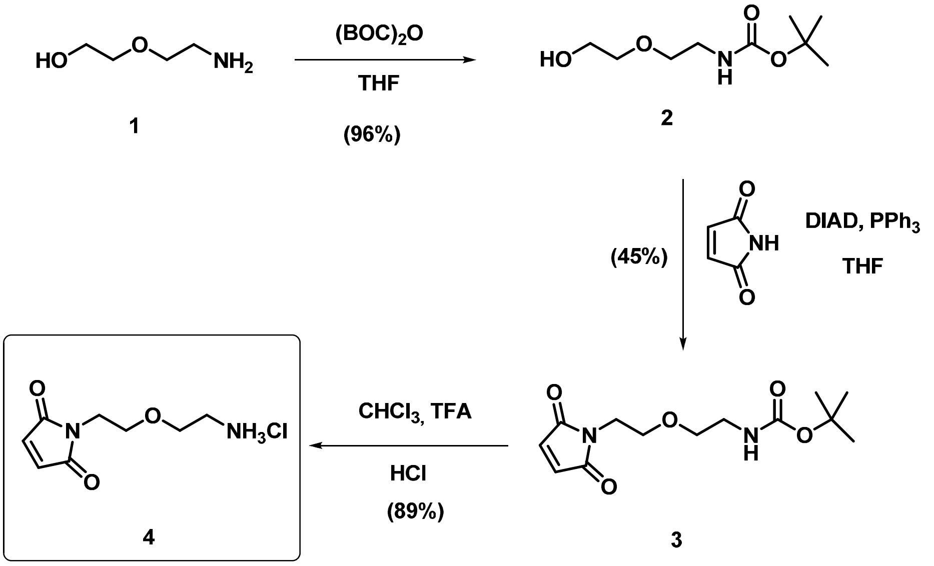

Scheme S1. Synthesis of the maleimido linker.

To a solution of **1** (2 mL, 18 mmol) in THF (16 mL), a solution of $(BOC)_2O$ (3.92 g, 18 mmol) in THF (16 mL) was added and stirred at room temperature for 16 h. Then, the solvent was evaporated and the residue was solved in CH_2Cl_2 and washed with brine, to obtain compound **2** as colorless oil in 96% yield. ^{1}H-NMR (300 MHz, $CDCl_3$) δ 3.83–3.68 (m, 2H), 3.64–3.47 (m, 4H), 3.33 (dd, *J* = 10.4, 5.2 Hz, 2H), 1.45 (s, 9H).

Over a solution of **2** (1.84 g, 9 mmol) and maleimide (2.1 g, 22.5 mmol) in THF (15 mL), a freshly prepared solution of PPh_3 (3.6 g, 13.5 mmol) and DIAD (3.6 mL, 18 mmol) in THF was added and stirred for 16 h. Then, the solvent was evaporated and abundant Et_2O was added. The solid appeared was removed by filtration and the filtrate was purified by flash chromatography (eluent: Hexane/AcOEt 5:2 to 1:1), to obtain compound **3** as white solid in 45% yield. ^{1}H NMR (300 MHz, CDCl3) δ 6.71 (s, 2H), 3.72 (t, *J* = 5.4 Hz, 2H), 3.60 (d, *J* = 5.4 Hz, 2H), 3.49 (t, *J* = 5.2 Hz, 2H), 3.26 (dd, *J* = 10.2, 5.2 Hz, 1H), 1.44 (s, 9H).

Over a solution of **3** (1.7 g, 5.98 mmol) in CH_3Cl (20 mL), trifluoroacetic acid (6 mL) was added and stirred at room temperature for 3 h. Then, the volatiles were evaporated. The oil obtained was dissolved in AcOEt and treated with an excess of HCl 10%. The aqueous phase was evaporated to obtain compound **4** as oil which solidified slowly in 89% yield. ^{1}H NMR (300 MHz, CDCl3) δ 8.23 (bs, 3H), 6.74 (s, 2H), 3.82–3.66 (m, 6H), 3.24–3.23 (m, 2H).

2. Titration curves of the interaction between TBAs and α-thrombin by UV–Visible

Calibration curves between TBA1 and TBA2 nanoparticles or O^6-MeG-TBA1 and TBA2 nanoparticles with increasing concentrations of α-thrombin protein (0×, 0.5×, 1×, 1.5× and 2×) were monitored by measuring changes in the UV spectrum (recorded from 650 to 400 nm). A mixture of NP–TBA1 and NP–TBA2 were diluted in buffer containing 10 mM phosphate (pH = 7) and 5 mM KCl to reach a concentration of 5 nM of nanoparticles in a volume of 500 μL. As conjugation was

approximated to represent a coating of 100 oligonucleotides per nanoparticle, the concentration of DNA in the sample was considered to be around 500 nM. UV of the sample without α-thrombin was recorded as negative control. Then, increasing concentrations of α-thrombin were added and after a quick manual mix, the UV spectra were recorded. The UV spectra of the mixture were recorded until reaching a final concentration of 1 µM of protein, which represents a 2:1 molar ratio between α-thrombin and DNA. This same experiment was repeated using the modified NP–methyl TBA1 and NP–TBA2. All spectra were overlaid together to study the displacement of the maximum peak from 520 nm in case of AuNPs and 545 nm for AuSPIONs to higher wavelengths, due to the formation of a network of TBAs-α-thrombin complexes. The protocol used for these studies was the same for AuNPs and for AuSPIONs.

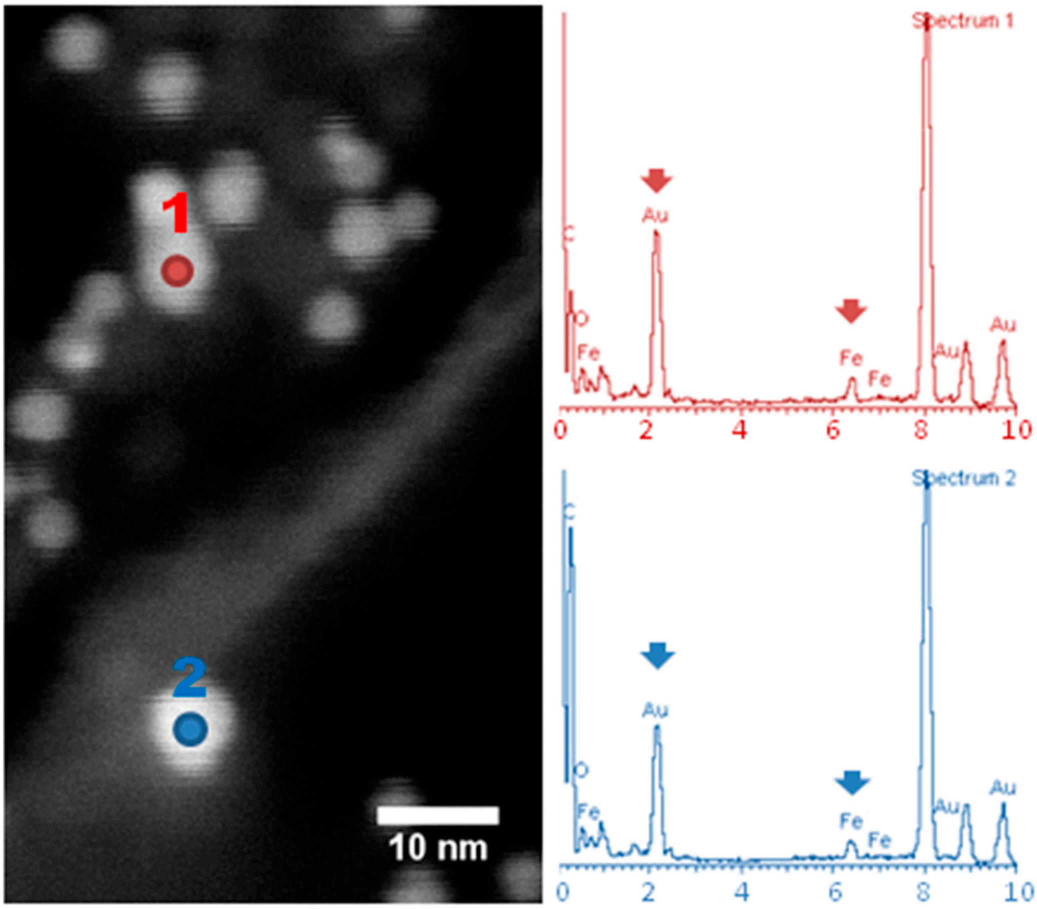

Figure S1. STEM micrograph (on the right) and related EDX spectrum of two selected AuSPIONs.

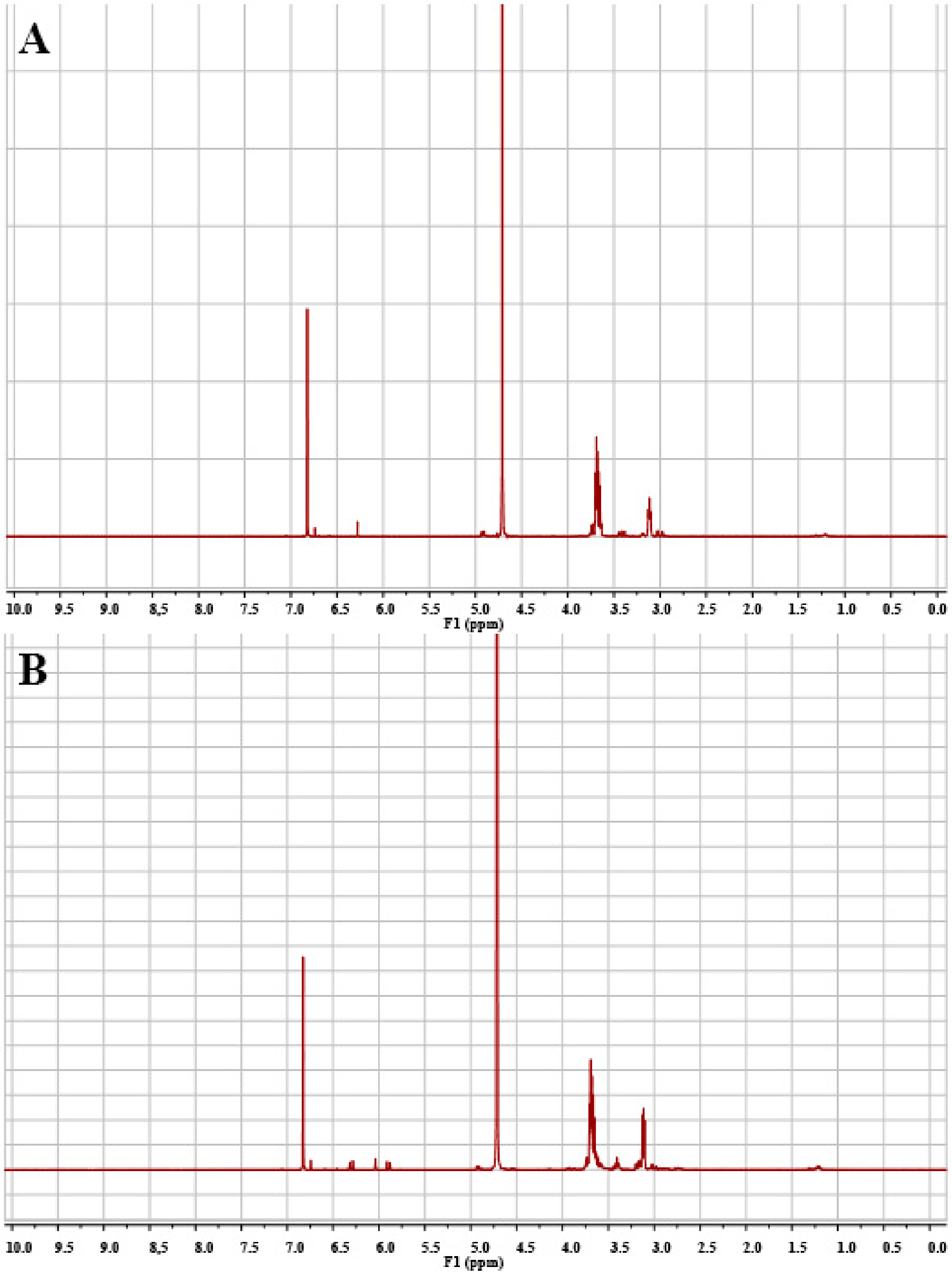

Figure S2. Stability of maleimide linker in D_2O during conjugation in $NaHCO_3$ solutions. (**A**) ^{1}H-NMR spectra of the maleimide linker after 16 h in D_2O solution; (**B**) ^{1}H-NMR spectra of the maleimide linker after 16 h in solution D_2O with 1 equiv. of $NaHCO_3$.

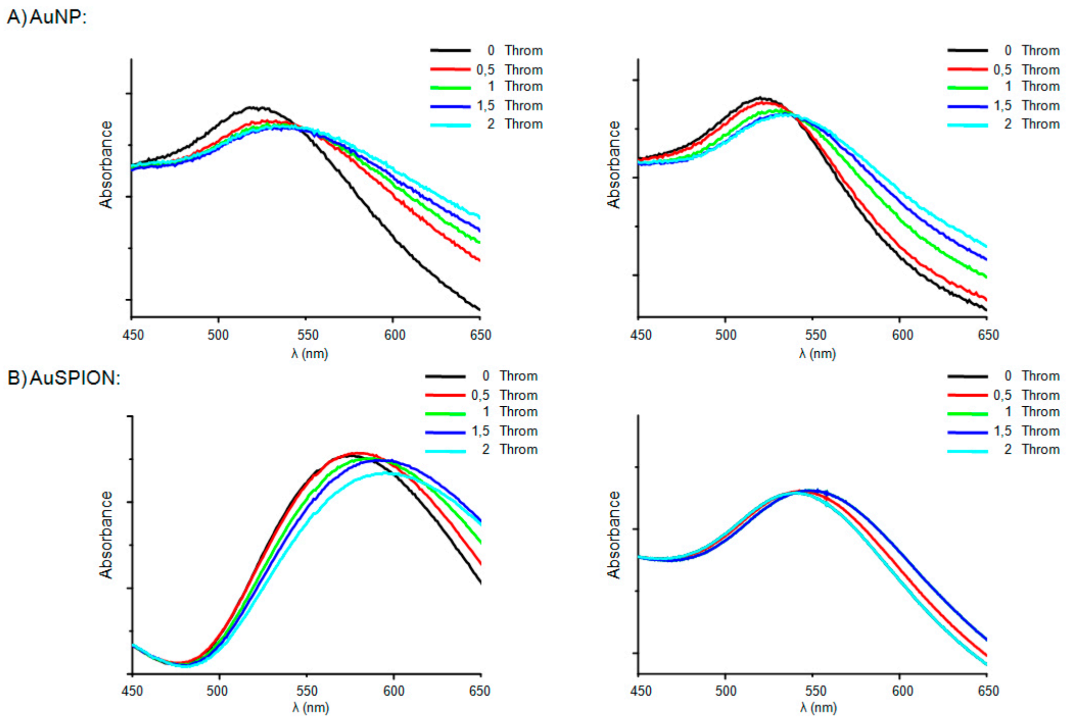

Figure S3. UV-Visible calibration curves. Spectra of the interaction between NPs-TBAs and α-thrombin upon increasing concentration of the protein (0×, 0.5×, 1×, 1.5× and 2×). Mixture of TBA1 and TBA2 NPs (**left** side) and O^6-MeG-TBA1 and TBA2 (**right** side). (**A**) AuNPs; and (**B**) AuSPION.

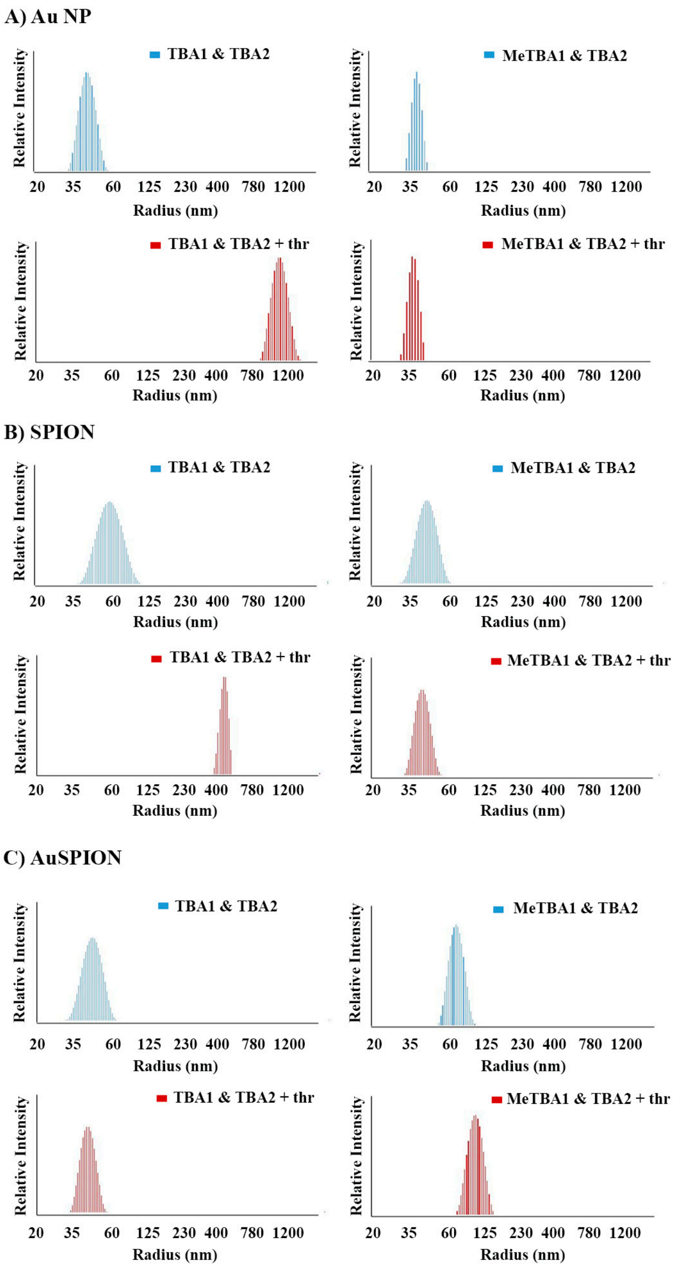

Figure S4. Particle size distribution of NPs-TBAs by DLS. Mixture of TBA1 and TBA2 NPs (**left** side) and O^6-MeG-TBA1 and TBA2 (**right** side) as a control are displayed in blue. The same NPs mixtures in the presence of α-thrombin are represented in red, below the control experiment. In all the experiments the molar ratio of α-thrombin:TBAs was 0.5:1. From top to bottom (**A**) AuNPs, (**B**) SPIONs and (**C**) AuSPION.

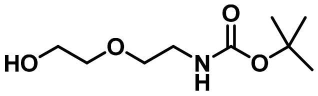

2

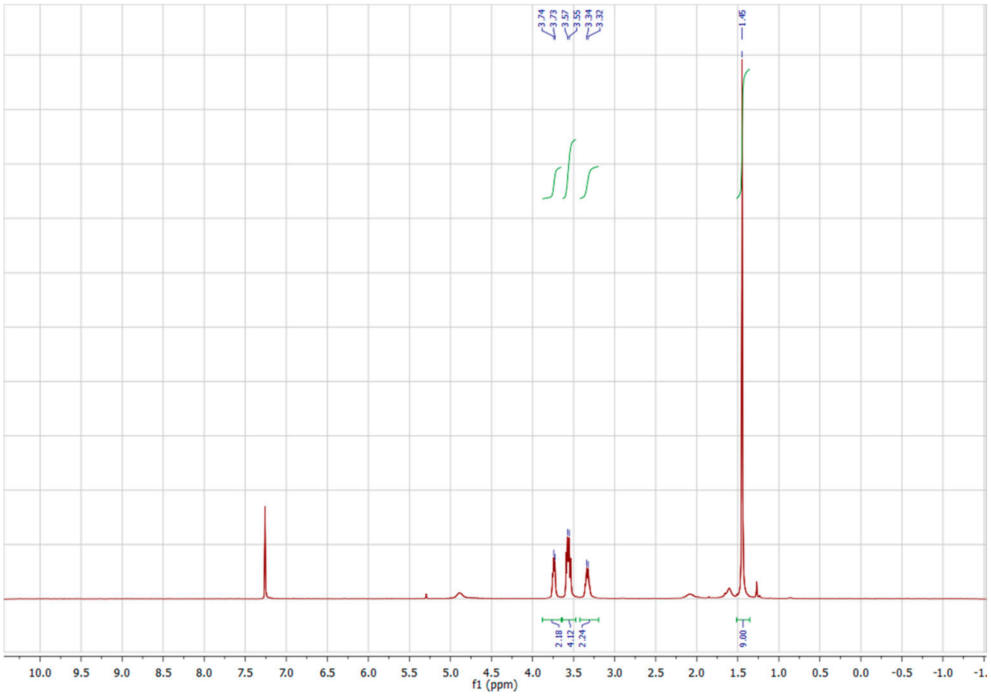

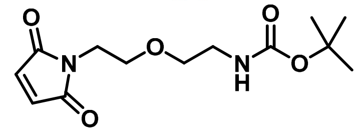

3

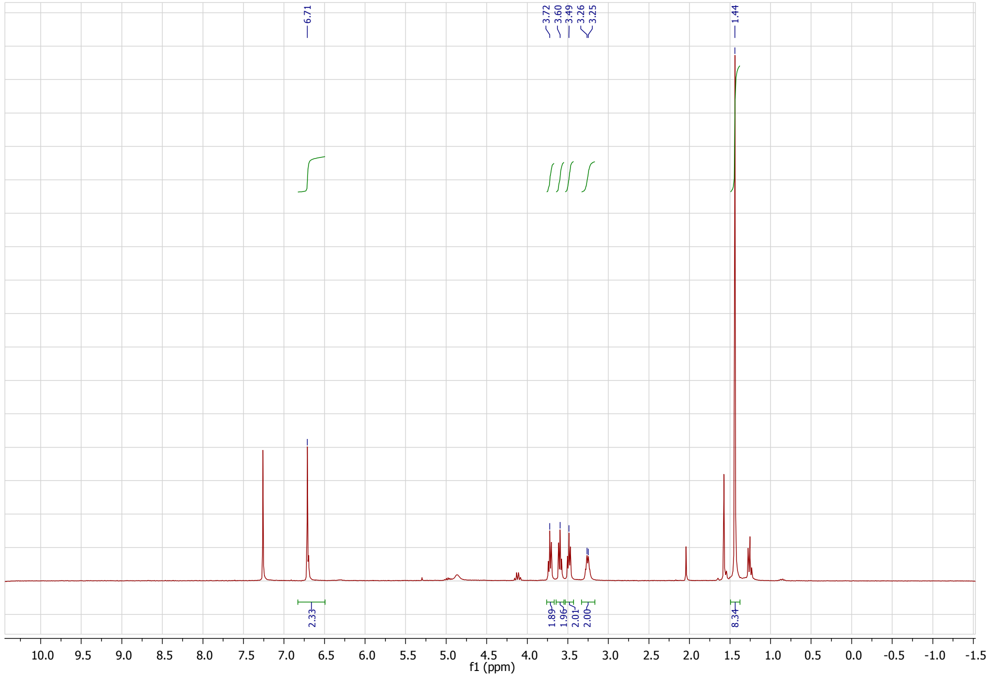

Figure S5. *Cont.*

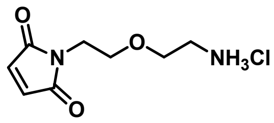

4

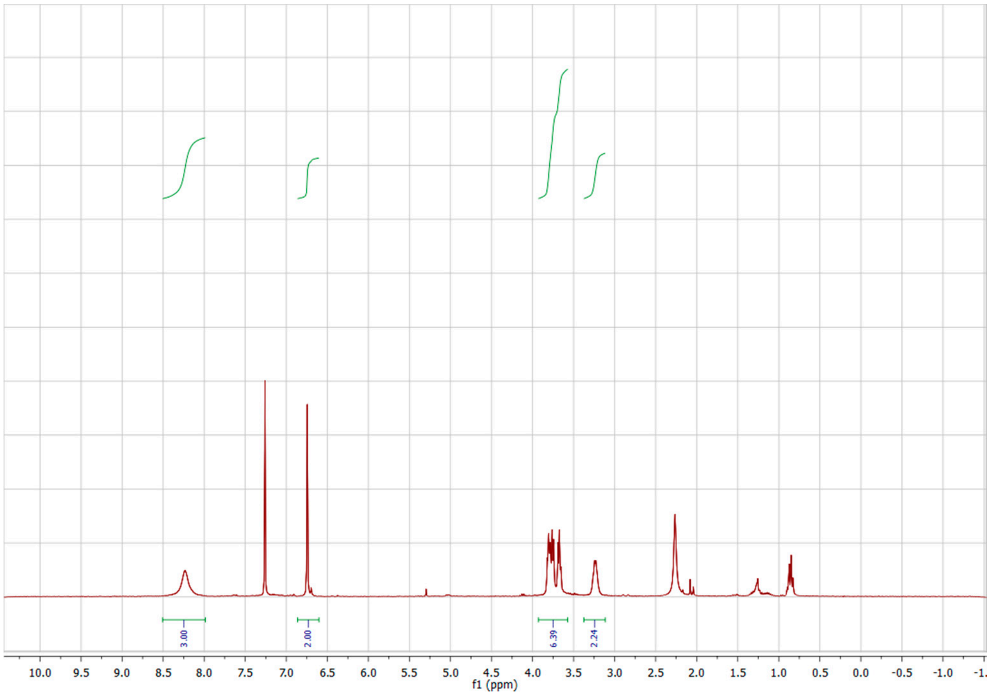

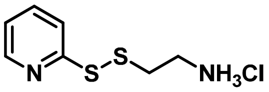

5

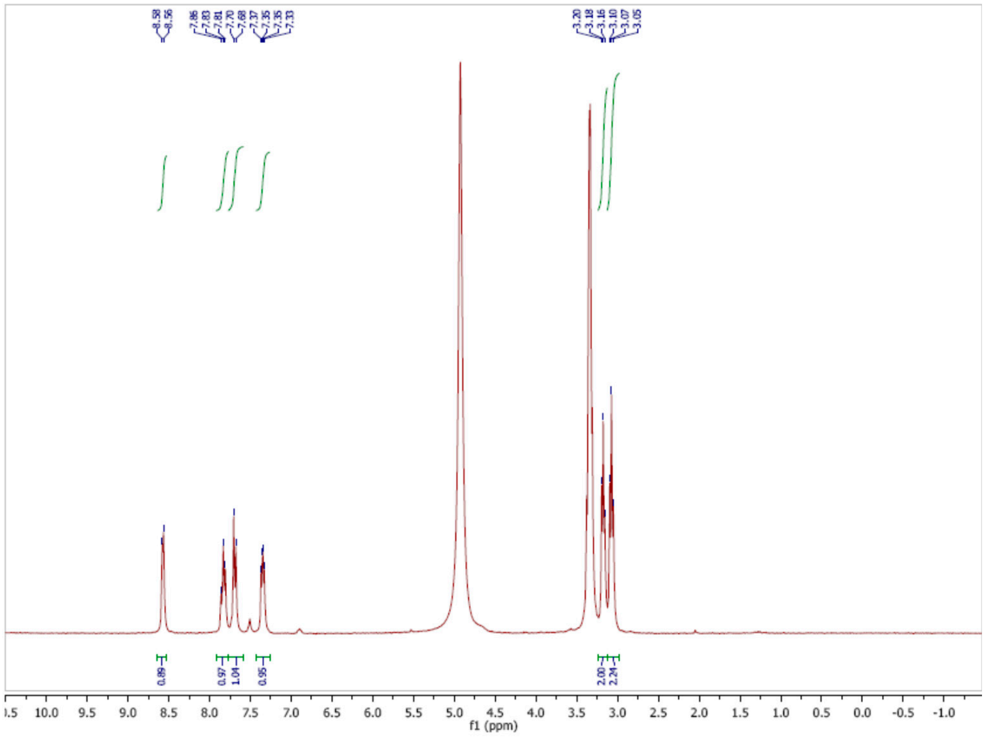

Figure S5. ^{1}H-NMR spectra of compounds **2**–**5**.
